# Bat Distribution Size or Shape as Determinant of Viral Richness in African Bats

**DOI:** 10.1371/journal.pone.0100172

**Published:** 2014-06-24

**Authors:** Gaël D. Maganga, Mathieu Bourgarel, Peter Vallo, Thierno D. Dallo, Carine Ngoagouni, Jan Felix Drexler, Christian Drosten, Emmanuel R. Nakouné, Eric M. Leroy, Serge Morand

**Affiliations:** 1 Centre International de Recherches Médicales de Franceville, Franceville, Gabon; 2 Institut National Supérieur d'Agronomie et de Biotechnologies (INSAB), Franceville, Gabon; 3 CIRAD, UPR AGIRs, Montpellier, France; 4 CIRAD, UPR AGIRs, Harare, Zimbabwe; 5 Institute of Vertebrate Biology, Academy of Sciences of the Czech Republic, Brno, Czech Republic; 6 Institute of Experimental Ecology, Ulm University, Ulm, Germany; 7 Institute of Virology, University of Bonn Medical Centre, Bonn, Germany; 8 Institut Pasteur de Bangui, Bangui, République Centrafricaine; 9 Institut de Recherche pour le Développement, UMR 224 (MIVEGEC), IRD/CNRS/UM1, Montpellier, France; 10 Institut des Sciences de l'Evolution, CNRS-UM2, CC065, Université de Montpellier 2, Montpellier, France; 11 Centre d'Infectiologie Christophe Mérieux du Laos, Vientiane, Lao PDR; CSIRO, Australia

## Abstract

The rising incidence of emerging infectious diseases (EID) is mostly linked to biodiversity loss, changes in habitat use and increasing habitat fragmentation. Bats are linked to a growing number of EID but few studies have explored the factors of viral richness in bats. These may have implications for role of bats as potential reservoirs. We investigated the determinants of viral richness in 15 species of African bats (8 *Pteropodidae* and 7 microchiroptera) in Central and West Africa for which we provide new information on virus infection and bat phylogeny. We performed the first comparative analysis testing the correlation of the fragmented geographical distribution (defined as the perimeter to area ratio) with viral richness in bats. Because of their potential effect, sampling effort, host body weight, ecological and behavioural traits such as roosting behaviour, migration and geographical range, were included into the analysis as variables. The results showed that the geographical distribution size, shape and host body weight have significant effects on viral richness in bats. Viral richness was higher in large-bodied bats which had larger and more fragmented distribution areas. Accumulation of viruses may be related to the historical expansion and contraction of bat species distribution range, with potentially strong effects of distribution edges on virus transmission. Two potential explanations may explain these results. A positive distribution edge effect on the abundance or distribution of some bat species could have facilitated host switches. Alternatively, parasitism could play a direct role in shaping the distribution range of hosts through host local extinction by virulent parasites. This study highlights the importance of considering the fragmentation of bat species geographical distribution in order to understand their role in the circulation of viruses in Africa.

## Introduction

Bats are linked to a growing number of emerging infectious diseases (EID) [1], [2] such as Ebola or Marburg Haemorrhagic fevers [3]–[5], SARS Coronavirus [6] and the newish Middle East respiratory syndrome coronavirus (MERS-CoV) [7]. This trend is, *inter alia*, linked to biodiversity loss, changes in habitat use and increased habitat fragmentation [8].

Few studies have investigated parasite species richness in bats [9]–[11]. However, Turmelle and Olival [12] showed viral richness in bats correlates with IUCN status and population genetic structure. The distribution range of hosts has been often considered as a potential determinant of parasite species richness [13]–[15]. Hosts distributed over large areas are more likely to encounter new parasites that may infect them [14], [16]. However, the shape of the distribution has received little attention [12], [13] but may have implications on the role of bats as pathogen reservoirs. Distribution shape and habitat fragmentation were observed at two different scales and Fahrig [17] suggested that the processes affecting changes in distribution and habitat preference of a species are independent. The shape of the distribution being mostly the products of speciation, extinction and range expansion [18]. Area shape is an important aspect of the distribution of animals and plants, which is strongly linked to population demographics and the subsequent contraction and expansion of their distribution [19], [20]. Therefore, area shape must be taken into account together with phylogenetic information in any comparative analysis of parasite diversity. Two alternative explanations can be proposed on the potential link between host distribution shape and parasite species richness: a longer border, due to fragmentation, may entail higher habitat diversity which would intensify contacts with various sources of parasites leading an overall increase in parasite diversity. Alternatively, a longer border may increase host species vulnerability due to area fragmentation and reduced host population size, hence pathogen transmission.

The first comparative analysis was performed to test the hypothesis that distribution shape and more specifically the fragmentation of the distribution area, correlates with viral richness in bats. We investigate the determinants of viral richness in 15 species of African bats, on which we found new information on virus infection and bat phylogeny. Body weight, roosting behaviour and migration [10], [21] were also included in our analysis because of their potential influences on parasite or viral species richness.

## Materials and Methods

### Ethic statements

All the capture events, animal handling, euthanasia and transfer of samples across country borders were performed in accordance with the guidelines of the American Society of Mammalogists (http://www.mammalsociety.org/committees/animal-care-and-use) [22]:

Bats were captured following recommendations by Kunz and Parsons [23]. Captured bats were removed carefully from nets as soon as possible to minimize injury, drowning, strangulation, or stress. Safe and humane euthanasia was achieved through the use of inhalant anaesthetic (halothane) prior to autopsy.

All work (capture, euthanasia and autopsy) was carried out with authorization from the respective wildlife authorities of each country. Capture and sacrifice Permit in Gabon: N°0021/MEFE- PA/SG/DGEF/DCF (2009) and N°0031/MEFDD/SG/DGEF/DFC (2010 and 2011), and from the Direction de la Faune et de la Chasse, Ministère des eaux et forêts, de l'environnement et du développement durable, Gabon. Capture and sacrifice permit in Central African Republic (CAR): N°038/MENAESR/D.CAB/DGESR/DRS/SCGPRS. 08, and from the Ministère de l'Education Nationale, de l'Alphabétisation, de l'Enseignement Supérieur et de la Recherche, CAR. Sample collection in Senegal and Republic of Congo: we used samples collected by previous studies on filovirus in bat populations [4], [24], [25].

### Study animals

**Figure 1:**
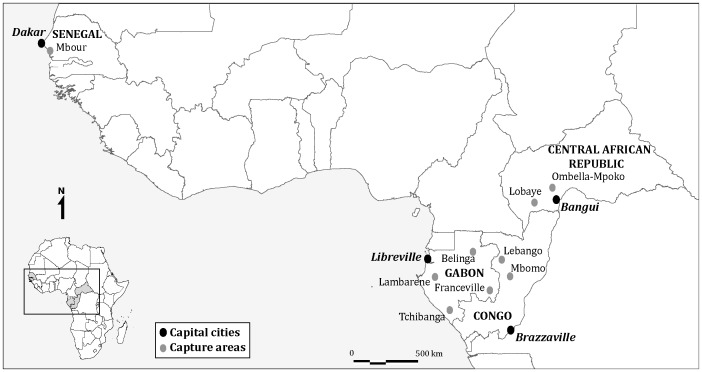
Geographic location of field sites where bats were captured.

Our study on the correlation of viral richness in bats was conducted using 15 bats species from Central and West Africa. We selected only the species for which we had enough samples and information on viral richness to carry out analysis. Bats were caught in the Republic of Congo, Gabon, Central African Republic (CAR) and Senegal [4]. In the Republic of Congo, bats were caught in 2005 and 2006 at Mbomo (0°25N; 14°41E) and Lebango (0°39′ N; 14°21′ E). In Gabon captures occurred at four sites in 2005, 2006, 2009 and 2010: the first one was located near Franceville (1°37S; 13°36E) the largest town of the Haut-Ogooué province in south-eastern Gabon; the second site was located close to Lambarene (0°41S; 11°01E), the largest town of the Moyen-Ogooué province in western Gabon; the third one was near Tchibanga (2°51S; 11°01E), the main town of the Nyanga province in south-western Gabon; and 3 caves (Faucon Cave: 1°07 N; 13°20 E, Zadié Cave: 0°98 N; 13°19 E and Batouala Cave: 0°82 N; 13°45 E) situated in the Belinga Mountain in Northeastern Gabon. In CAR, samples were collected in 2008 and 2009 at 3 localities: Lobaye (3°46′ S; 18°34′ E), Ombella-Mpoko (4°33′ S; 18°30′ E), and Bangui (4°21 N; 18°33 E), the capital. In Senegal, captures took place at Mbour in 2006 (14°25′ N; 16°57′ E) located about 80 km from Dakar, capital of Senegal (Figure 1).

Bats were captured using mist-nets or harp traps. Mist-nets (12×2.4 m) were hoisted either in the tree canopy (defined as “foliage”) or at the entrance of the small roosting caves (defined as “cave”) just before twilight. Harp Traps were used at the entrance of big caves known to harbor large population of bats. Following capture, bats were identified on site by trained field biologists and individually euthanized under sedation in a field laboratory. Bats were weighed using a spring scale prior to autopsy and selected internal organs were collected during autopsy and stored at −80°C for future virological analysis. Data on the ecological traits of the 15 different bat species captured (i.e., roost type, body weight, migratory behaviour and colony size) was gathered from published literature (Table 1, see Annex 1 for references).

**Table 1:** Factors tested as potential determinants of viral richness (References in Annexe 1).

Bats species	Viral Richness*	Sample size	Geographical range (km^2^)	Fragmen-tation	Roost type**	Body weight (g)***	Migratory	Colony size
*Coleura afra*	1	85	3,573,000	0.86	Cave	9.9	Yes	1000
*Eidolon helvum*	12	1019	14,510,000	0.23	Foliage	177.3	Yes	500000
*Epomophorus gambianus*	4	169	4,947,000	0.34	Cave	87.2	Yes	50
*Epomops franqueti*	4	763	4,564,000	0.28	Foliage	114.7	Yes	5
*Hipposideros* cf. *ruber*	4	585	8,056,000	0.57	Cave	8.2	Yes	500000
*Hipposideros gigas*	5	230	4,357,000	0.39	Cave	109	Yes	300–1000
*Hypsignathus monstrosus*	5	188	3,562,000	0.53	Foliage	312.5	Yes	25–132
*Megaloglossus woermanni*	1	49	3,498,000	0.38	Foliage	13.3	Yes	-
*Micropteropus pusillus*	5	706	6,704,000	0.37	Foliage	26.1	No	1–10
*Miniopterus inflatus*	3	275	2,423,000	0.53	Cave	9.5	Yes	50
*Mops condylurus*	4	446	9,355,130	0.30	Cave	22.45	Yes	18–200
*Myonycteris torquata*	3	580	4,624,000	0.29	Foliage	45.7	Yes	-
*Neoromicia tenuipinnis*	0	35	4,279,511	0.41	Cave	5.3	Yes	20
*Rousettus aegyptiacus*	13	1828	4,989,000	0.91	Cave	120.3	No	5000
*Taphozous mauritianus*	0	9	12,436,000	0.23	Foliage	27.8	No	12

*Viral richness is obtained from the number of individual bats that we have sampled combined with animals sampled as reported in published papers.

**Foliage includes bats that roost in trees: main bough, under bark, within foliage, hollow branches, under exposed roots, deep in dense foliage and in tree trunks. Cave includes tunnels, cavities or crevices, abandoned mine shafts, roofs and basements of houses.

***Average body weight, both sexes combined.

### Bat phylogeny

In order to improve the quality of the comparative analysis, a phylogenetic tree was built using 14 new molecular sequences of the bat mitochondrial cytochrome *b* gene (Table 2). Total genomic DNA was extracted from ethanol-preserved tissue samples (muscle, liver or spleen) with Genomic DNA Tissue Mini Kit (Geneaid Biotech) according to the manufacturer's protocol. We amplified the mitochondrial gene for cytochrome *b* (*cytb*) using primer pairs F1 (modified; 5′- CCACGACCAATGACAYGAAAA-3′) and R1 from Sakai et al. [26] in most microbats, L14724 and H15915 from Irwin et al. [27] in hipposiderids and fruit bats, LGL765F and LGL766R from Bickham et al. [28], [29] in long-fingered bats (*Miniopterus inflatus*). The volume of PCR reaction was 25 µl, it contained 12.5 µl Combi PPP Master Mix (Top-Bio, Prague, Czech Republic), 200 µM of forward and reverse primers respectively, and 2.5 µl of extracted DNA. PCR protocol consisted in an initial denaturation at 94°C for 3 min, 35 cycles of denaturation for 40 s at 94°C, annealing for 40 s at 50°C, and extension for 90 s at 65°C, and a final extension at 65°C for 5 min. Resulting PCR products were inspected on 1.5% agarose gel and purified with Gel/PCR DNA Fragments Extraction Kit (Geneaid Biotech). If multiple bands appeared, the one of appropriate length was excised and purified from gel using the same purification kit. Purified PCR products were sequenced commercially (Macrogen, Seoul, Korea) with the respective forward primer using BigDye Terminator sequencing chemistry (Applied Biosystems, Foster City, CA, USA) on ABI 3730xl sequencer. Sequences were edited in Sequencher 4.6 (Gene Codes, Ann Arbor, MI, USA), manually checked for correct base reading and protein coding frame, and aligned by eye in BioEdit 7.0 [30]. Sequences of two artiodactyl taxa, *Bos taurus* (D34635) and *Ovis ammon* (AJ867276) were added to the alignment as outgroup taxa for rooting the bat phylogeny. Phylogenetic tree including branch lengths was inferred from aligned nucleotide sequences in PAUP*4.0b (Sinauer Associates, Sunderland, Massachusetts, USA) under maximum likelihood (ML) criterion and general time-reversible model of evolution with a portion of invariable sites and gamma distributed variation rates (GTR+I+Γ), which was suggested as the best evolutionary model and whose parameters were estimated in Modeltest 3.7. Topological constraints were set before computation of the ML tree, as corresponding to acknowledged phylogenetic relationships among genera, families and higher taxonomic ranks of bats as referred by Teeling et al. [31] and Almeida et al. [32]. Due to *a priori* definition of the tree topology, analysis of nodal support was not performed. The constrained ML tree was, however, compared to unconstrained ML tree using a Shimodaira-Hasegawa (SH) test, in order to assess possible significant difference, which might indicate unreliability of the constrained tree. Sequences generated in this study were deposited in the EMBL/DDBJ/Genbank databases under accession number (JQ956436-JQ956449).

**Table 2:** Characteristic of bats included into phylogenetic analyses in this study and accessions number for all *cytB* sequences.

Sample ID	Year of collection	Bat species	Sex	Country	Locality	Tissue source	Source	GenBank accession no.
GB2139	2005	*Megaloglossus woermanni*	M	Congo	Mbomo	Liver	CIRMF	JQ956436
GB2225	2005	*Myonycteris torquata*	M	Congo	Lebango	Liver	CIRMF	JQ956437
09/760	2009	*Micropteropus pusillus*	F	RCA	Ombella-Mpoko	Spleen	IP Bangui	JQ956438
GB2569	2006	*Hypsignathus monstrosus*	F	Congo	Mbomo	Spleen	CIRMF	JQ956439
GB1961	2005	*Epomops franqueti*	M	Congo	Lebango	Spleen	CIRMF	JQ956440
GB1661	2005	*Eidolon helvum*	F	Gabon	Lambaréné	Spleen	CIRMF	JQ956441
GB3320	2006	*Epomophorus gambianus*	M	Senegal	Mbour	Liver	CIRMF	JQ956442
GB0685	2009	*Hipposideros gigas*	M	Gabon	Belinga	Spleen	CIRMF	JQ956443
08/316	2008	*Taphozous mauritianus*	M	RCA	Lobaye	Spleen	IP Bangui	JQ956444
08/207	2008	*Mops condylurus*	M	RCA	Lobaye	Liver	IP Bangui	JQ956445
08/322	2008	*Neoromicia tenuipinnis*	M	RCA	Lobaye	Spleen	IP Bangui	JQ956446
GB0332	2009	*Miniopterus inflatus*	M	Gabon	Belinga	Patagium	CIRMF	JQ956447
GB0675	2009	*Hipposideros* cf. *ruber*	F	Gabon	Belinga	Liver	CIRMF	JQ956448
GB0415	2009	*Coleura afra*	M	Gabon	Belinga	Patagium	CIRMF	JQ956449

IP: Institut Pasteur; No data available for the sequence of *Rousettus aegyptiacus* (Genbank accession number AB085740).

### Viral richness

Two methods were used to document viral richness of the studied bat species. First, we tested our bat samples for viruses. We used (i) nested Reverse-Transcription polymerase chain reaction (RT-PCR) assay targeting the RNA-dependent RNA polymerase gene using generic consensus primers for the genus *Coronavirus* [33]; (ii) hemi-nested RT-PCR targeting the N terminal end of the NS5 gene by using degenerate primers for the genus *Flavivirus* [34], [35]; and (iii) filoviruses (Marburg virus and Ebola virus) as previously described [4], [36] (Table 3). Then, additional virological data were drawn from literature. In published papers, the methods used to detect viruses directly were mouse inoculation, cell culture, electron microscopy and PCR; indirect methods utilised to detect markers of replication and viral infection in bats from organs, tissues or blood were direct fluorescent antibody, indirect fluorescence antibodies, radio immuno assay, rapid fluorescent focus inhibition test, fluorescent antibody test, and seroneutralization. The serological detection of arbovirus antibodies alone (particularly genus *Flavivirus* and *Alphavirus*) was not considered as evidence of a viral association because of some degree of cross-reaction within the virus family, rendering it difficult to differentiate viruses. Viruses forming distinct clusters within the same genus were recorded as a unique viral species. For example, in *Rousettus aegyptiacus*, bat gammaherpes viruses (Bat GHV) 1, 2, 4, 5, 6 and 7 were recorded as one unique viral species and Bat GHV 3 as another viral species [37]. For Ebola virus, different viral species of this genus were considered as a single virus. For each bat species, we calculated the viral richness as the total number of different viruses described for the given bat species.

**Table 3:** Samples used for viral screening.

			*Coronavirus*		*Flavivirus*		*Marburg virus*		*Ebola virus*	
Sampling site	Species	Total of samples collected	N° of tested	N° of positive	N° of tested	N° of positive	N° of tested	N° of positive	N° of tested	N° of positive
Gabon	*Coleura afra*	31	23	0	29	0	-	-	31	0
	*Eidolon helvum*	60	48	0	32	0	-	-	-	-
	*Epomops franqueti*	498	358	0	140	0	-	-	-	-
	***Hipposideros*** cf.*ruber*	540	387	3	498	0	-	-	521	0
	***Hipposideros gigas***	234	228	0	227	1	-	-	233	0
	*Hypsignathus monstrosus*	43	40	0	14	0	-	-	1	0
	*Megaloglossus woermanni*	50	47	0	16	0	-	-	-	-
	*Micropteropus pusillus*	47	43	0	37	0	-	-	-	-
	*Miniopterus inflatus*	190	52	0	179	0	-	-	186	0
	*Myonycteris torqutata*	243	220	0	98	0	-	-	-	-
	*Rhinolophus cf. alcyone*	15	15	0	15	0	-	-	15	0
	***Rousettus aegyptiacus***	582	492	0	305	1	-	-	187	0
Congo	***Epomops franqueti***	393	286	0	128	2*	-	-	-	-
	*Hypsignathus monstrosus*	94	42	0	74	0	-	-	-	-
	*Megaloglossus woermanni*	20	5	0	20	0	-	-	-	-
	*Micropteropus pusillus*	273	129	0	100	0	-	-	-	-
	*Myonycteris torquata*	589	286	0	136	0	-	-	-	-
	*Rousettus aegyptiacus*	5	2	0	5	0	-	-	-	-
Senegal	*Eidolon helvum*	32	18	0	-	-	-	-	-	-
	*Epomophorus gambianus*	15	15	0	-	-	-	-	-	-
	*Rousettus aegyptiacus*	58	-	-	-	-	-	-	-	-
RCA	*Eidolon helvum*	295	295	0	295	0	295	0	295	0
	*Epomophorus gambianus*	19	19	0	19	0	19	0	19	0
	*Epomops franqueti*	81	81	0	81	0	81	0	81	0
	*Hipposideros gigas*	2	2	0	2	0	2	0	2	0
	*Hypsignathus monstrosus*	28	28	0	28	0	28	0	28	0
	*Megaloglossus woermanni*	3	3	0	3	0	3	0	3	0
	***Micropteropus pusillus***	533	533	2*	533	0	533	0	533	0
	*Mops condylurus*	160	160	0	160	0	160	0	160	0
	*Myonycteris torquata*	12	12	0	12	0	12	0	12	0
	*Neoromicia tenuipinnis*	4	4	0	4	0	4	0	4	0
	*Rousettus aegyptiacus*	1	1	0	1	0	1	0	1	0
	*Taphozous mauritianus*	8	0	0	8	0	8	0	8	0

*Pools of ten each.

### Geographical distribution size and shape

**Figure 2:**
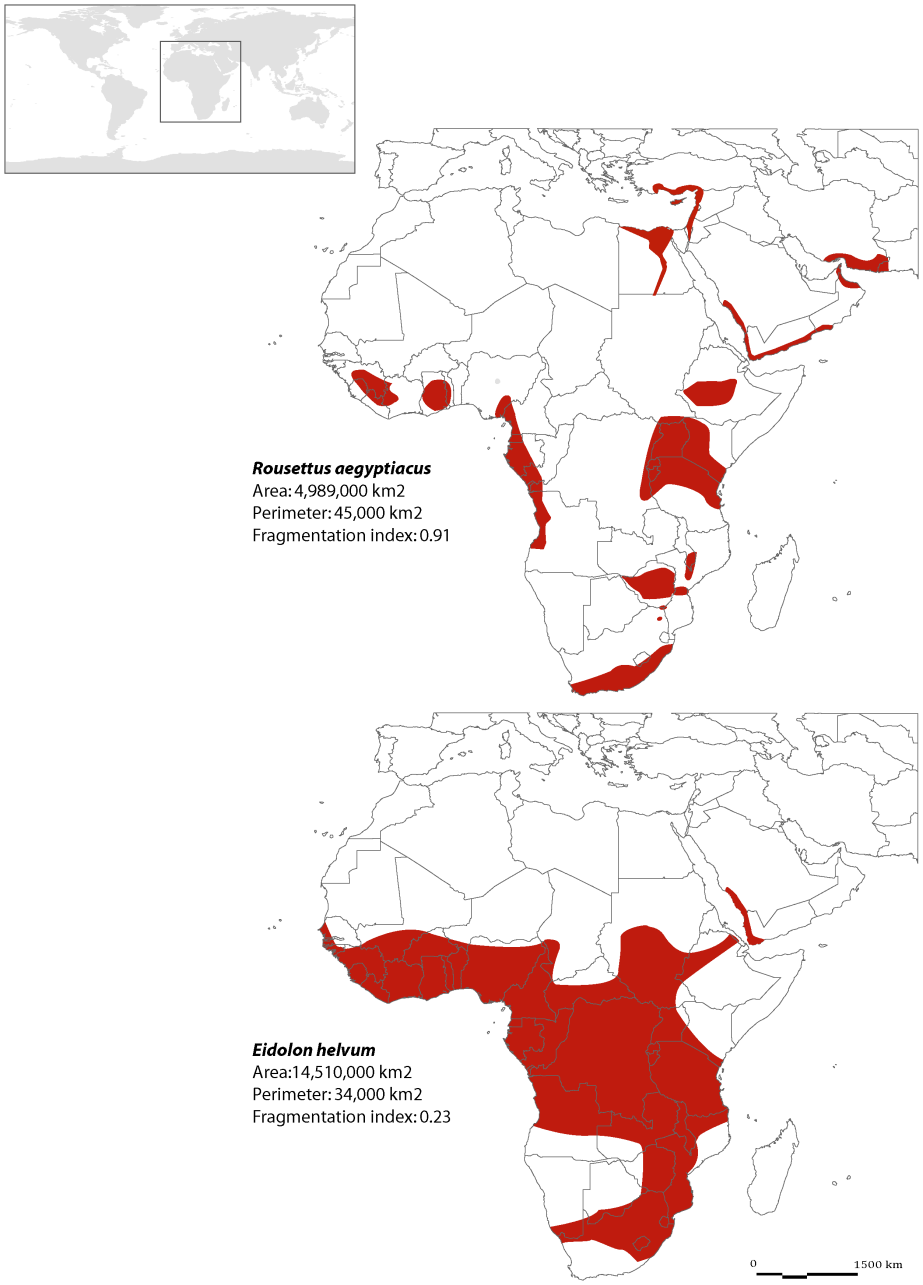
Two examples of bat geographical distribution showing contrasted distribution shape or fragmentation (from [69]).

To test the impact of the fragmentation of the distribution area on viral richness in bats, we used the geographic range maps of each studied bat species provided by the ‘IUCN Red List of Threatened Species’ web site, one of the biggest databases available on mammalian distribution, based on international experts' knowledge. The maps were imported in a GIS using MapInfo professional V 5.5. We then drew polygons following species distribution to obtain area and perimeter measures for all drawn polygons. The shape of the geographic range was estimated using the ratio of the total perimeter to the total surface area following the approach used by Kauffman cited in Fortin et al. [38]. The higher the ratio, the greater is the fragmentation of the distribution (Figure 2).

### Comparative analyses of the determinants of viral richness

Using information on bat phylogeny described above, we calculated the independent contrasts for each of the investigated variables with the package APE [39] implemented in R (R Development Core Team 2013). To confirm the proper standardization of contrasts, we regressed the absolute values of standardized contrasts against their standard deviations. Contrasts were then analysed using standard multiple regressions, with all intercepts forced through the origin [40]. We tested the importance of the phylogenetic signal on each variable using the parameter K (which is the ratio of observed phylogenetic covariance divided by the expected covariance under Brownian motion), with the package picante [41] implemented in R (R Development Core Team 2013).

As in previous studies [12], [13], we performed standard multiple regressions using independent contrasts, with the intercept forced at zero and viral richness as the dependent variable. Independent variables were geographical range, fragmentation of the distribution, roost type (foliage *vs* cave), average body weight and migratory behaviour (yes *vs* no) (Table 1). We did not include colony size as variable as information was missing for two species. Number of sampled hosts or sampling effort (number of samples we tested added to the number of samples reported in published papers) ware also considered as an independent variable. The analysis was conducted on 14 of the 15 captured species for which sample size was considered sufficient (>30). We then selected the best subset selection of variables using AIC criteria.

## Results

### Viral richness

We detected coronaviruses from *Hipposideros cf. ruber* (accession numbers JX174638-JX174640) and *Micropteropus pusillus* (JX174641 and JX174642). Flaviviruses were detected from *Rousettus aegyptiacus* (JX174643), *Hipposideros gigas* (JX174644) and *Epomops franqueti* (JX174645 and JX174646) (Table 3). We compiled our results with the data found in the literature. We found information on viruses for the 15 selected bat species except for *Neoromicia tenuipinnis* and *Taphozous mauritianus* (Table 4).

**Table 4:** List of viruses found in this study and completed with data from the literature.

Species	Virus	References
*Eidolon helvum*	Lagos bat virus (LBV), Mokola virus, West Caucasian (WC) virus, Zaire Ebola virus (ZEBOV), Ife virus (Orbivirus), Hendra virus, Nipah virus (NPHV), Rubulavirus, Coronavirus, Rotavirus related, Simplexvirus, Parvovirus	[44]–[56]
*Micropteropus pusillus*	LBV, Coronavirus, ZEBOV, Marburg virus (MBGV), Rift Valley Fever virus (RVF)	This study; [4], [57], [58]
*Rousettus aegyptiacus*	LBV, Bat Gammaherpesvirus (1, 2, 4, 5, 6, 7), Bat Gammaherpesvirus 3, Betaherpesvirus, MBGV, Coronavirus, ZEBOV, Yogue virus, Kasokero virus, Chiropteran Papillomavirus, Henipavirus, Rubulavirus, Flavivirus	This study; [4], [5], [36],
*Miniopterus inflatus*	MBGV, Coronavirus, Rubulavirus	[48], [54], [60], [61]
*Hipposideros* cf. *Ruber*	RVF, Rubulavirus, Morbillivirus unclassified, Coronavirus,	This study; [54], [58], [64]
*Hipposideros gigas*	Rubulavirus, Morbillivirus unclassified, Flavivirus, Shimoni bat virus, SARS-like CoV	This study; [54], [62], [65]
*Epomops franqueti*	ZEBOV, Reston Ebola virus, MBGV, Flavivirus	This study; [2], [4], [24], [66]
*Coleura afra*	Morbillivirus unclassified	[54]
*Myonycteris torquata*	ZEBOV, Coronavirus (SARS-CoV), Henipavirus	[2], [4], [24], [54], [61], [66]
*Hypsignathus monstrosus*	ZEBOV, Reston Ebola virus, MBGV, Coronavirus (SARS-CoV), NPHV	[2], [4], [24], [45], [46], [54], [61], [66]
*Megaloglossus woermanni*	Rubulavirus	[54]
*Neoromicia tenuipinnis*	No virus found	
*Taphozous mauritianus*	No virus found	
*Mops condylurus*	Bukalassa bat virus, Dakar bat virus, Entebbe bat virus, Coronavirus (SARS-CoV)	[61], [67], [68]
*Epomophorus gambianus*	LBV, NPHV, ZEBOV, Reston Ebola virus	[45], [46], [52], [66]

West, East and Central Africa, Europe (species from zoo, unspecified origin), South Africa, USA (species from zoo, unspecified origin).

### Bat Phylogeny

**Figure 3:**
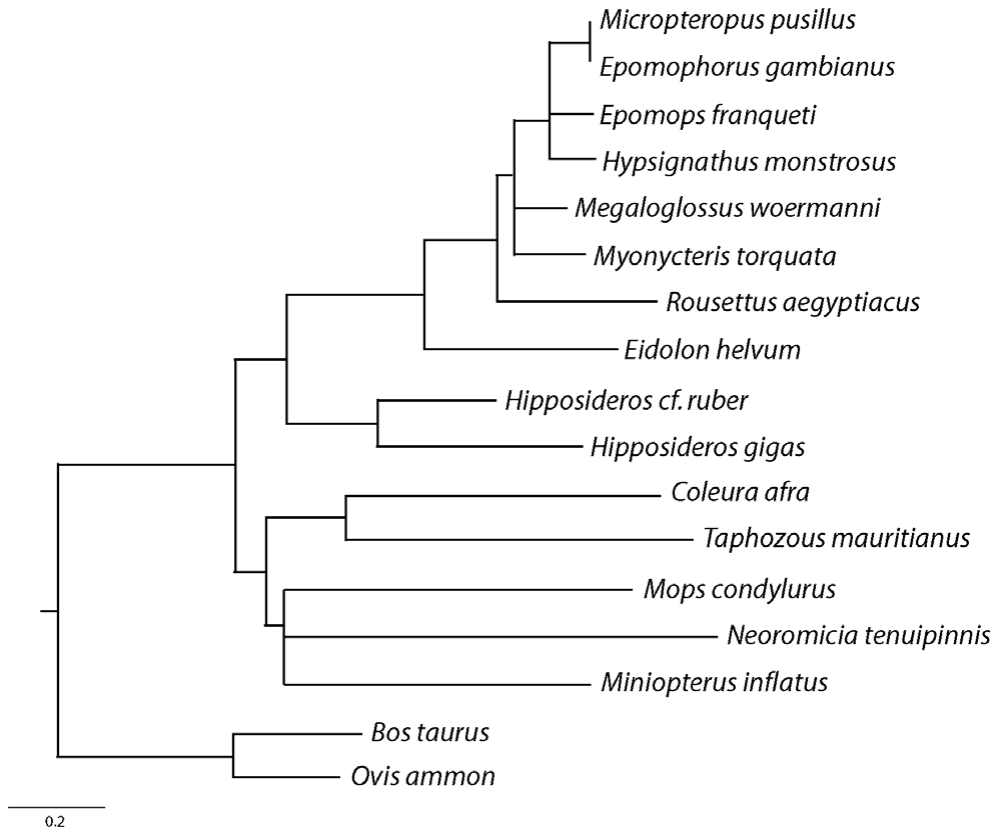
Phylogeny of the African bat species investigated in this study.

We reconstructed the phylogenetic tree of the bat species investigated in this analysis using 15 sequences under the constraint of acknowledged taxonomic relationships (Figure 3). The constrained tree (−lnL = 6439.91045) did not differ significantly from the unconstrained tree (SH test: diff. lnL = 7.89267, P = 0.126), and was thus considered as a reasonable depiction of bat phylogeny.

### Determinant of the viral richness

Only viral richness showed statistically significant level of phylogenetic signal using estimates of K among all the traits investigated (Table 5). However, distribution shape showed a level of phylogenetic close to significance (Table 5).

**Table 5:** Levels of phylogenetic signal in the variables investigated using the parameter K and the parameter lambda.

Variables	K	P (no signal)
Viral richness	0.519	0.044
Host sample size	0.071	0.529
Host weight (body weight)	0.089	0.433
Distribution size	0.164	0.302
Distribution shape	0.474	0.072
Roosting site	0.023	0.478
Migration	0.014	0.732

**Figure 4:**
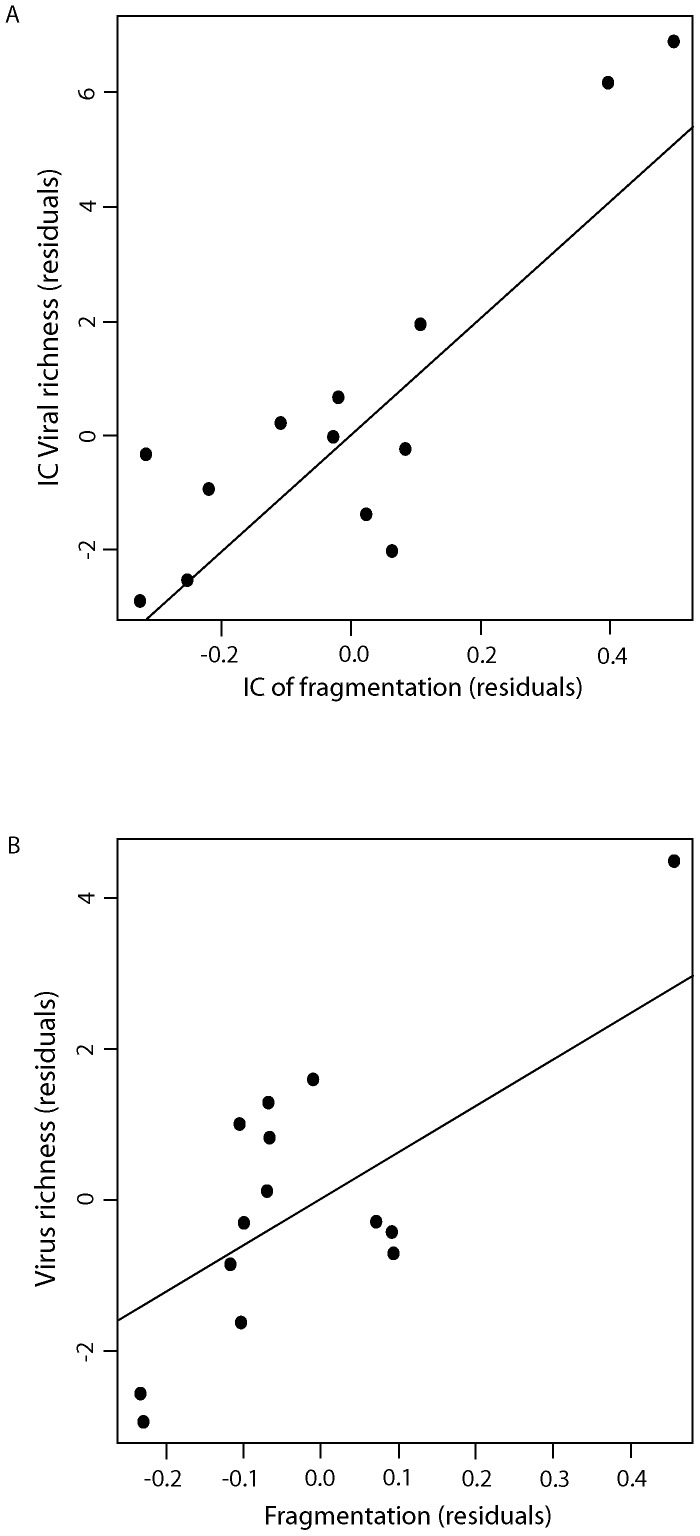
Partial relationship between viral richness and distribution fragmentation, assessed by a measure of distribution shape using (A) phylogenetic independent contrasts, or (B) raw values (and using residuals from the general regression modelling in **Table 7**).

Four variables were retained in the preferred model, which was back-selected, based on the AIC criterion, and using the raw data (non corrected for phylogeny) (Table 6). Using the independent contrasts (variables controlled for phylogeny), the best model had the same four independent variables (Table 6). Taking into account host sampling, we found that viral richness in bats was greater in large-bodied and widely distributed bats and when their geographical distribution was fragmented (Tables 5 & 6). There were no significant relationships between viral richness and migratory behaviour or roosting behaviour. Finally, greater fragmentation of the geographic distribution was highly associated with increased viral richness (Table 7, Figures 4A & 4B).

**Table 6:** Comparison of models used to test the effects of several independent variables (weight, size and shape of distribution, migration, roosting and sample size) on viral richness of bats (using the independent contrasts), using phylogenetic regression (Independent contrasts) or non-phylogenetic regression (raw values).

Analysis	Model ranks	AIC
Phylogenetic regression (Independent contrasts)	Weight + distribution size + distribution shape + sample size	19.93
	Weight + distribution size + distribution shape + roosting + sample size	20.67
	Weight + distribution size+ distribution shape + migration + roosting + sample size	22.66
Non-phylogenetic	Weight + distribution size + distribution shape + sample size	17.91
	Weight + distribution size + distribution shape + roosting + sample size	19.51
	Weight + distribution size+ distribution shape + migration + roosting + sample size	20.87

Models are ranked from the least to the most supported according to corrected Akaike information criteria (AIC).

**Table 7:** Best model explaining viral richness in bats using independent contrasts (initial model is given in Table 6), using the phylogenetic regression (independent contrasts) and non-phylogenetic regression (raw values' and independent variables are ranked according to their contributions to the models using F values).

Analysis	Independent variables	Slope (SD), P	*F*-test	P	R^2^,
					*F*-total (P)
Phylogenetic regression (Independent contrasts)	Distribution shape	10.25 (2.18), 0.001	35.8	0.0002	
	Host weight	3.12 (0.63), 0.0008	6.6	0.031	
	Host sample	1.59 (0.65), 0.037	5.9	0.03	
					R^2^ = 0.89
					F_4,9_ = 17.9
					(0.0003)
Non-phylogenetic	Host weight	2.82 (0.87), 0.009	31.95	0.0002	
	Distribution shape	6.71 (2.38), 0.02	12.66	0.005	
	Host sample	3.17 (0.78),0.002	16.51	0.002	
	Distribution size	0.001 (0.0001), 0.01	7.16	0.02	
					R^2^ = 0.87
					F_4,10_ = 17.1 (0.0002)

## Discussion

This is the first comparative analysis investigating the effect of distribution shape, i.e. geographical range fragmentation or edge range density, on viral richness in bats. Our first hypothesis was that bats living in caves in sympatry with other species with increased promiscuity and high population density of susceptible individuals, would generate opportunities for cross-species transmission of viruses and their rapid spread. However, our study does not support this hypothesis. Our results showed a significant influence of host body weight, distribution size and shape on viral richness; viral richness increases with larger distribution areas and fragmentation of bat distribution, according to the measure of their distribution shape. Before discussing this correlation, the difference between habitat fragmentation and habitat loss should be considered since Fahrig [17] suggested that the two processes are independent. An ecological explanation of the correlation between viral richness and distribution could be interpreted in the light of the historical biogeography of African bats, which falls within the domain of phylogeny and phylogeographic studies [31]. Range distributions and shapes are the product of speciation, extinction and historical displacements [18]. The accumulation of parasite species, viruses in the present study, could be related to the historical expansion and contraction of bat species' distribution ranges, with potentially strong effects of distribution edges on virus transmission. Indeed, the marginal effect of phylogenetic signal on the distribution shape of the investigated bats (Table 5) suggests that both history and current ecological drivers may have shaped their distribution. For a given distribution area, the most fragmented distributions contain more edges than the less fragmented ones. Positive edge effects could be responsible for the positive effects of distribution shape on either the abundance or distribution of some bat species that may have facilitated virus host switches. However, critical information to explore this issue further is lacking due to the limits of current knowledge on African bats' phylogeography as well as the geographic distribution and phylogeny of their viruses (such as bats and rabies-related viruses [42]). Furthermore, it should be noted that the use of the distribution area obtained from ICUN Red List might not accurately describe the distribution shape of bat species. More accurate and precise distributions would definitively improve the robustness of the study.

An alternative explanation produced by a theoretical study, attributes a direct role of parasitism in limiting the distribution range of hosts through the extinction of local hosts by virulent parasites [43]. However, this hypothesis has not been tested using empirical data.

As previously emphasized, we must differentiate the fragmentation of the distribution from habitat loss, as the consequences on bat species of the habitat loss are likely to be different to the consequences of the range fragmentation. Habitat loss following land use changes has been perceived as a major threat to biological diversity, whereas fragmentation may be positive or negative [42]. Habitat losses may increase species losses and, in turn, induce changes in ecosystem functions, including parasitism. Several studies have shown that parasites suffer more from habitat loss and isolation than their hosts, but other studies emphasize that habitat loss may increase the abundance of some hosts, and consequently their parasite loads, through an increase of host density-dependent transmission [13]. The consequences in terms of surveillance, spill-over and emergence in human populations are then species specific, in relation to their historical biogeography, actual range size and shape, and on-going loss of habitat. As already emphasized by Turmelle and Olival [12], while biogeography can help to identify macro-ecological determinants of pathogen richness, and potentially epidemiological processes, control strategies need to be carried out at local geographic scales.

The number of viruses found in bats in our study added to the viruses described in bats in the literature is certainly an underestimation. Indeed, bats are reservoirs for many viruses and have the peculiarity to maintain viral replication at relatively low levels. Thus, chronicity of viral infections in bats requires the use of highly sensitive detection tools. However, in our study, samples were tested by Reverse-Transcription PCR assay using generic consensus primers, known to decrease sensitivity. The detection of these viruses may be improved by more sensitive methods, such as high-throughput sequencing and viral isolation yet much more expensive than PCR.
